# Association of Chronic Obstructive Pulmonary Disease With Arrhythmia Risks: A Systematic Review and Meta-Analysis

**DOI:** 10.3389/fcvm.2021.732349

**Published:** 2021-09-30

**Authors:** Xin Liu, Zhuohui Chen, Siyuan Li, Shuo Xu

**Affiliations:** ^1^Department of Critical Care Medicine, The First Affiliated Hospital of Gannan Medical University, Ganzhou, China; ^2^Second Clinical Medical College, Nanchang University, Nanchang, China; ^3^Department of Pulmonary and Critical Care Medicine, The Ganzhou People's Hospital, Ganzhou, China

**Keywords:** chronic obstructive pulmonary disease (COPD), arrhythmia, risk factor, meta-analysis, atrial fibrillation

## Abstract

**Background:** A large number of studies have shown that the arrhythmia risks may be the potential causes of death among chronic obstructive pulmonary disease (COPD) patients. However, the association of COPD with risks of arrhythmias has never been systematically reviewed. Therefore, we performed a meta-analysis to assess the relationship between COPD and arrhythmia risks.

**Methods:** An updated systematic retrieval was carried out within the databases of Embase and PubMed until June 27, 2021.The random-effects model was used to pool studies due to the potential heterogeneity across the included studies. The risk ratios (RRs) with 95% confidence intervals (CIs) were regarded as effect estimates.

**Results:** A total of 21 studies were included in our meta-analysis. In the pooled analysis by the random-effects model, the results showed that COPD was significantly related to the risk of atrial fibrillation (AF) (RR = 1.99, 95% CI: 1.46–2.70), ventricular arrhythmias (VA) (RR = 2.01, 95% CI: 1.42–2.85), and sudden cardiac death (SCD) (RR = 1.68, 95% CI: 1.28–2.21). The corresponding results were not changed after exclusion one study at a time. The pooled results were also stable when we re-performed the analysis using the fixed-effects model.

**Conclusions:** Our current data suggested that COPD was associated with increased risks of AF, VA, and SCD.

## Introduction

Chronic obstructive pulmonary disease (COPD) is a chronic condition and imposes a heavy burden on both public health and the social economy. Available evidence (1) indicates that the worldwide incidence rate of COPD is estimated at 4–5 million cases per year, and nearly 10% of adults aged 40 years or older suffer from COPD. Moreover, the mortality rate of COPD has been increasing during the past few years. Not only COPD itself but also COPD-related complications account for a sizable proportion of the rising mortality, and one-third of deaths are associated with cardiovascular diseases (2). Indeed, COPD patients are more likely to develop cardiovascular diseases such as arrhythmia, ischemic heart disease, [200mm][-13mm] Q10heart failure, pulmonary circulation disease, and arterial disease (3). In addition, COPD and cardiovascular diseases share common risk factors such as smoking and obesity (4). Systemic inflammatory states in patients with COPD can aggravate the development of atherosclerosis, giving rise to the risk of heart failure (5).

Recent studies (6, 7) have shown COPD may be responsible for the development of arrhythmias. A comprehensive study conducted by Rusinowicz et al. (8) has shown that COPD exacerbation is related to a high incidence of arrhythmias, and COPD patients receiving conventional therapy can increase the risk of arrhythmias. Rusnak et al. (9) pointed out that COPD patients, who were presented with ventricular arrhythmias (VA), were associated with a higher rate of all-cause mortality. The lower forced expiratory volume at 1 s (FEV_1_) and forced vital capacity (FVC) are related to a higher risk of atrial fibrillation (AF) (10). In addition, COPD is found to be associated with a high risk of sudden cardiac death (SCD) in the community (11). Researching on the associations of COPD with the arrhythmia risks is deemed important since it may have implications for the management of these patients. In a word, the relationship between COPD and arrhythmia has not been fully elucidated and high-quality evidence is needed. The objective of this study is to prove the association between COPD and arrhythmia risks clearly, this allows medical personnel to adjust the treatment of patients with COPD.

Herein, we conducted a systematic review and meta-analysis to evaluate the relationship between COPD and arrhythmia risks among individuals without preexisting arrhythmias.

## Methods

Our current meta-analysis was performed following the Preferred Reporting Items for Systematic Reviews and Meta-Analyses guidelines (12). We did not need to provide ethical approval because only published articles were included in this meta-analysis. Readers can obtain data, methods, and materials by contacting the corresponding author to reproduce the results or the program.

### Retrieval Strategy

We systematically searched the databases of Embase and PubMed until June 27, 2021 for studies reporting the relationship between COPD and the development of AF, VA, and SCD. We applied the following keywords and searched terms to found out the relevant studies: (1) COPD OR chronic obstructive pulmonary disease OR lung function AND (2) arrhythmia OR atrial fibrillation OR ventricular tachycardia OR ventricular arrhythmia OR sudden cardiac death. To avoid missing the potential studies, the reference lists of the included studies were checked. No language restriction was applied in the searching process.

### Eligibility Criteria

We included studies that reported the relationship between COPD and the risk of AF, VA, and SCD with no restriction of study type. The definitions of COPD and outcomes (AF, VA, or SCD) were based on the original pieces of literature. Studies would be excluded if they had no data (e.g., comments, case reports, reviews, editorials, letters). Conference abstracts were also excluded because they did not have enough information to assess the study quality.

### Extraction Data

Two authors independently screened the studies from the databases. We then read the titles and abstracts of the records to select potential studies that could be used. And then, we screened the full text of these potential studies to identify the final studies. Discrepancies and disagreements were resolved by consensus or discussion with other authors.

For each included study, we extracted the following characteristics: the first author and publication year, study design, data source, information of population (age, sex, and sample size), definitions of COPD, and outcomes of AF, VA, or SCD, follow-up duration, and effect estimates. Adjusted risk ratios (RRs) and confidence intervals (CIs) were extracted from each included study.

### Quality Assessment

We assessed the study quality using the Newcastle-Ottawa Scale (NOS). The NOS tool included three major sections as follows: the selection of cohorts (0–4 points), the comparability of cohorts (0–2 points), and the assessment of the outcome (0–3 points). In this meta-analysis, a NOS score of ≥6 points was regarded as a moderate-to-high quality, while a NOS score of <6 points was regarded as a low-quality. The *post-hoc* analysis of RCT was regarded as a cohort study to conduct the quality assessment (13).

### Statistical Analysis

We assessed the heterogeneity across the included studies using the *I*^2^-values. In this study, *I*^2^ > 50% indicated significant heterogeneity. In the pooled analysis, we calculated the natural logarithm of the RR (Ln[RR]) and its standard error (SELn[RR]). The random-effects model was used to pool the Ln[RR] and SELn[RR] due to the potential heterogeneity across the included studies (14, 15). In the sensitivity analysis, we excluded one study at a time to examine the effect of each study on the pooled results. In addition, we used the fixed-effects model to re-perform the meta-analysis. The publication bias was checked by observing the symmetry characteristics of the funnel plots, a further assessed statistically using the Egger's and Begg's tests.

All statistical analyses were performed using the Stata software (version 15.0, Stata Corp LP, College Station, TX). In the pooled analysis, the statistical significance threshold was set at a *P*-value of < 0.05.

## Results

### Study Selection

**Figure 1:**
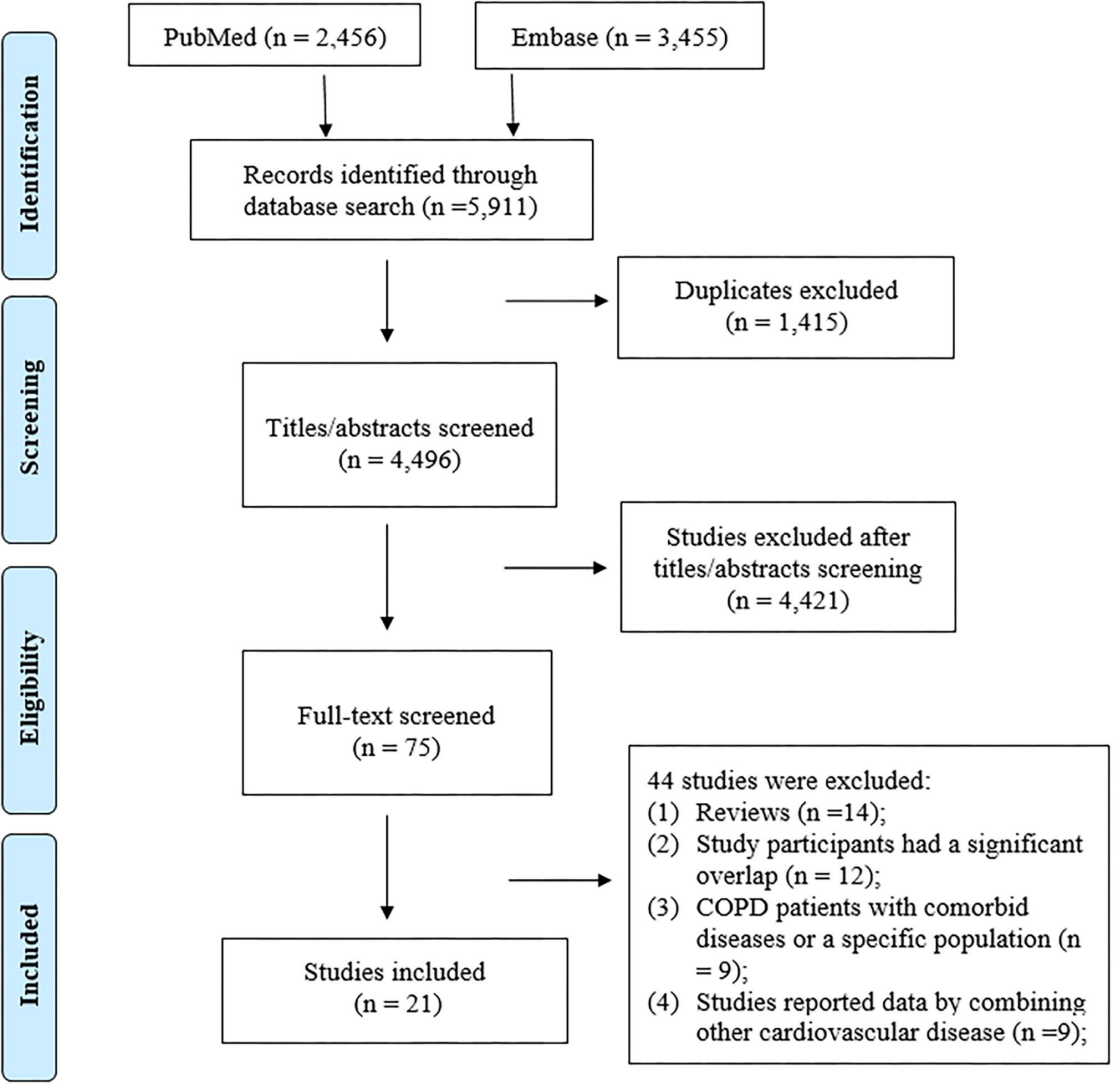
PRISMA flow diagram of the literature search process.

The process of the literature search is shown in Figure 1. A total of 21 studies (1 *post-hoc* analysis of RCT, and 20 observational studies) were included in our meta-analysis with a total of 7,916,582 participants (Supplementary Table 1).

The baseline characteristics of the included studies are illustrated in Table 1. These studies were published from 2002 to 2021, and the sample size ranged from 103 to 2,499,235 participants. Among the 21 included studies, 14 were prospective studies and 7 were retrospective studies. A total of 11, 8, and 5 studies provided the effect estimates for the risks of AF, VA, and SCD, respectively. The definition of COPD, AF, VA, and SCD in included studies were listed in Supplementary Tables 2–5. All the included studies had a moderate-to-high quality with a NOS score ranging from 6 to 8 points. The result of enrolled studies used the multivariable regression models (adjusting for confounders including age, sex, left atrial size, cardiovascular diseases, smoke) or propensity score-based analytical methods (balance baseline patient characteristics) (Supplementary Table 6).

**Table 1:** Baseline information of the selected studies in meta-analysis.

**Included study**	**Country**	**Study type**	**Data source**	**Sample Size**	**Study population characteristics**	**Whether use bronchodilator**	**Age (years)**	**Males (%)**	**Follow-up (year)**	**NOS**
Jingjing Li et al. (16)	USA	Prospective/Longitudinal study	4 US communities	15,004	People with special diseases (atherosclerosis)	NA	54	44.9	17.5	8
Douglas W. Mapel et al. (17)	USA	Prospective/Longitudinal study	Austin Automation Center	948,633	Normal	NA	66	98.1	7	7
Matthew Knuiman et al., (18)	Australia	Prospective/Longitudinal study	Electoral Registers for the Busselton district Busselton	4,843	Normal	NA	54	NA	15	6
Gregory Y.H. Lip et al. (19)	Denmark	Prospective/Longitudinal study	Danish Civil Registration System	2,499,235	Normal	NA	70	47	16.9	7
Yan Guang Li et al. (20)	France	Retrospective/Longitudinal study	A national hospitalization database in France	240,459	People with special diseases (post-ischemic stroke)	NA	73.52	52.4	0.66	8
Li et al. (21)	Asia	Retrospective/Longitudinal study	Chinese Yunnan Insurance Database	922,665	Normal	NA	47	52.8	7.2	8
Carter et al., (22)	USA	Prospective/Longitudinal study	7 NHS hospitals across the North West of England	2,968,182	Normal	NA	70.1	51	5.2	7
Maxim Grymonprez et al. (23)	Netherland	Prospective/Longitudinal study	Netherlands Organization for Scientific Research	10,943	Normal	NA	66.5	42.5	8.8	7
Liang et al., (24)	China	Retrospective/*Post-hoc* analysis of RCT	Treatment of preserved cardiac function heart failure with an aldosterone antagonist	2022	People with special diseases (heart failure)	NA	67.01	45.3	NA	8
Buzea et al., (25)	Romania	Prospective/Longitudinal study	University of Medicine and Pharmacy “Carol Davila”; Clinical Hospital Colentina	111	Normal	NA	62.78	56.8	15	8
Kumar Narayanan et al. (11)	USA	Prospective/Cross-sectional study	The Heart Institute, Cedars-Sinai Medical Center, Los Angeles, California	1,276	Normal	SBAs;	68.55	65.2	NA	6
Tomas Konecny et al. (26)	USA	Retrospective/Longitudinal study	Mayo Clinic Laboratory	6,351	Normal	NA	66	52.0	10	8
Tomas Konecny et al. (27)	USA	Retrospective/Longitudinal study	Mayo Clinic Institutional Review Board	7,441	Normal	Beta-agonistic bronchodilator	64	51.0	9	7
Lies Lahousse et al. (28)	Netherland	Prospective/Longitudinal study	Ommoord area of Rotterdam	13,471	Normal	NA	64	41.8	24	7
Zhi-Bin Kong et al. (29)	China	Prospective/Longitudinal study	Shanghai Sixth People's Hospital east campus	196	Normal	Bronchodilator	67.54	70.4	3	7
Sidney et al., (30)	USA	Retrospective/Longitudinal study	California state hospitalization with a primary hospital	91,932	Normal	NA	64.4	55.4	3	8
Yuji Kusunoki et al. (7)	Japan	Prospective/Longitudinal study	Nippon Medical School in Tokyo	103	Normal	NA	68.8	87.4	3.1	8
Chen et al., (31)	China	Retrospective/Longitudinal study	National Health Insurance Research Database	143,676	Normal	SABAs	57.66	54	12	8
Urena et al., (32)	Canada	Prospective/Longitudinal study	18 centers in North, America, South America, and Europe	3,726	People with special diseases (valvular heart disease)	NA	81	50.2	1.83	7
Nishiyama et al., (33)	Japan	Prospective/Longitudinal study	30 institutions in Japan	9,877	People with special diseases (coronary heart disease)	NA	67.5	71.4	3.56	7
Al-Khatib et al., (34)	Netherland	Prospective/*Post-hoc* analysis of RCT	a combined randomized trials database	26,436	Normal	NA	66	67.0	0.5	6

*RCT, randomized controlled trial; NA, not available*.

### Relationship Between COPD and AF Risk

**Figure 2:**
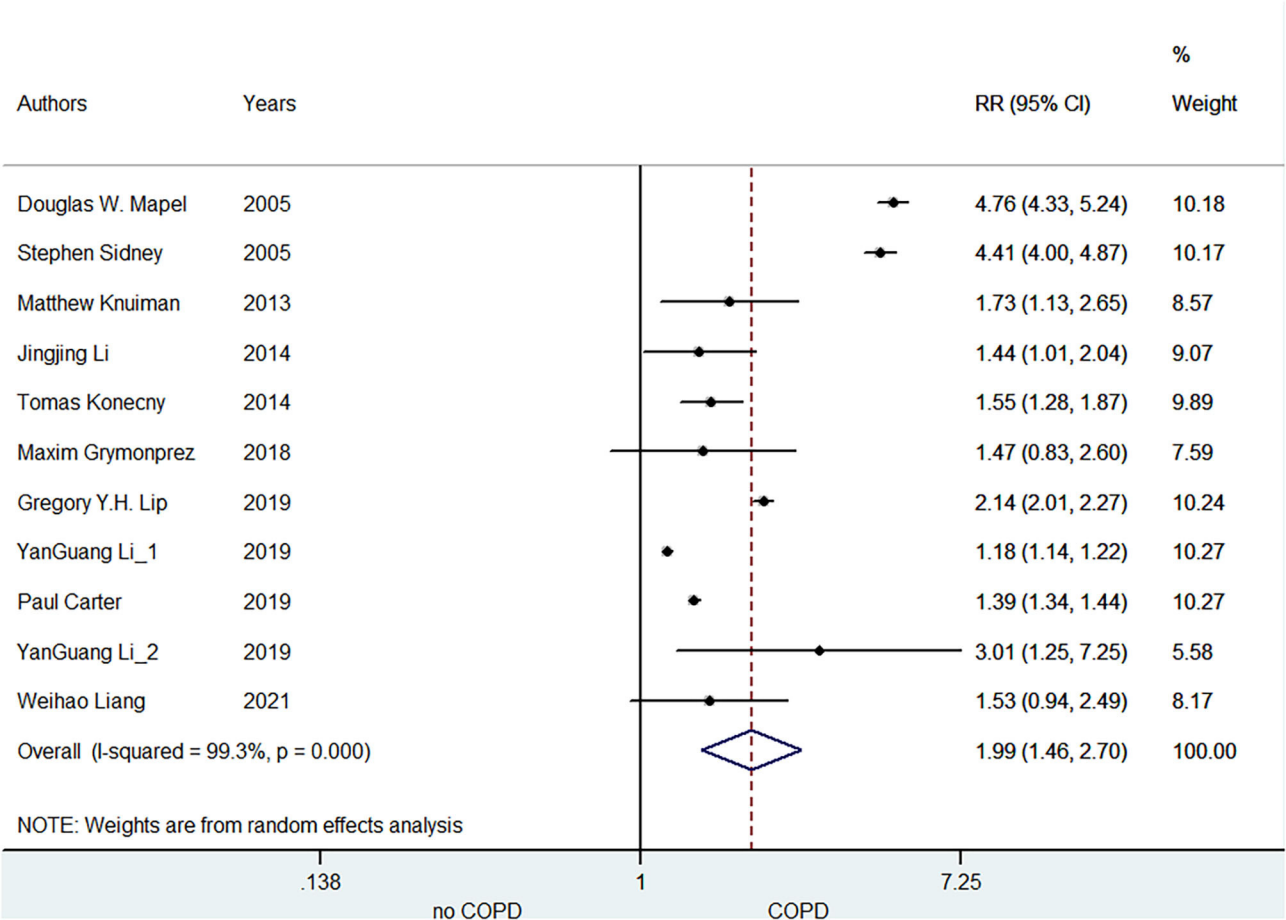
Meta-analysis of the pooled risk ratio of AF in COPD patients. AF, atrial fibrillation; RR, risk ratio.

A total of 11 included studies reported the association of COPD with AF risk. Li (16) reported the race- and sex-specific incidence rates of AF for airflow obstruction, separately. Lip et al. (19) reported the relationship between COPD and AF incidence at three distinct age cohorts (65, 70, and 75 years), separately. Grymonprez (23) studied this association according to the exacerbation frequency of COPD, and Konecny (27) reported the association between the severity of COPD and arrhythmias. Mapel et al. (17) provided the outcomes of AF and atrial flutter, respectively. Therefore, we combined the separate data of these studies as the final effect estimates in the pooled analysis. As shown in Figure 2, the pooled result from the random-effects model showed that COPD was associated with an increased risk of AF (RR = 1.99, 95% CI:1.46–2.70).

### Relationship Between COPD and VA Risk

**Figure 3:**
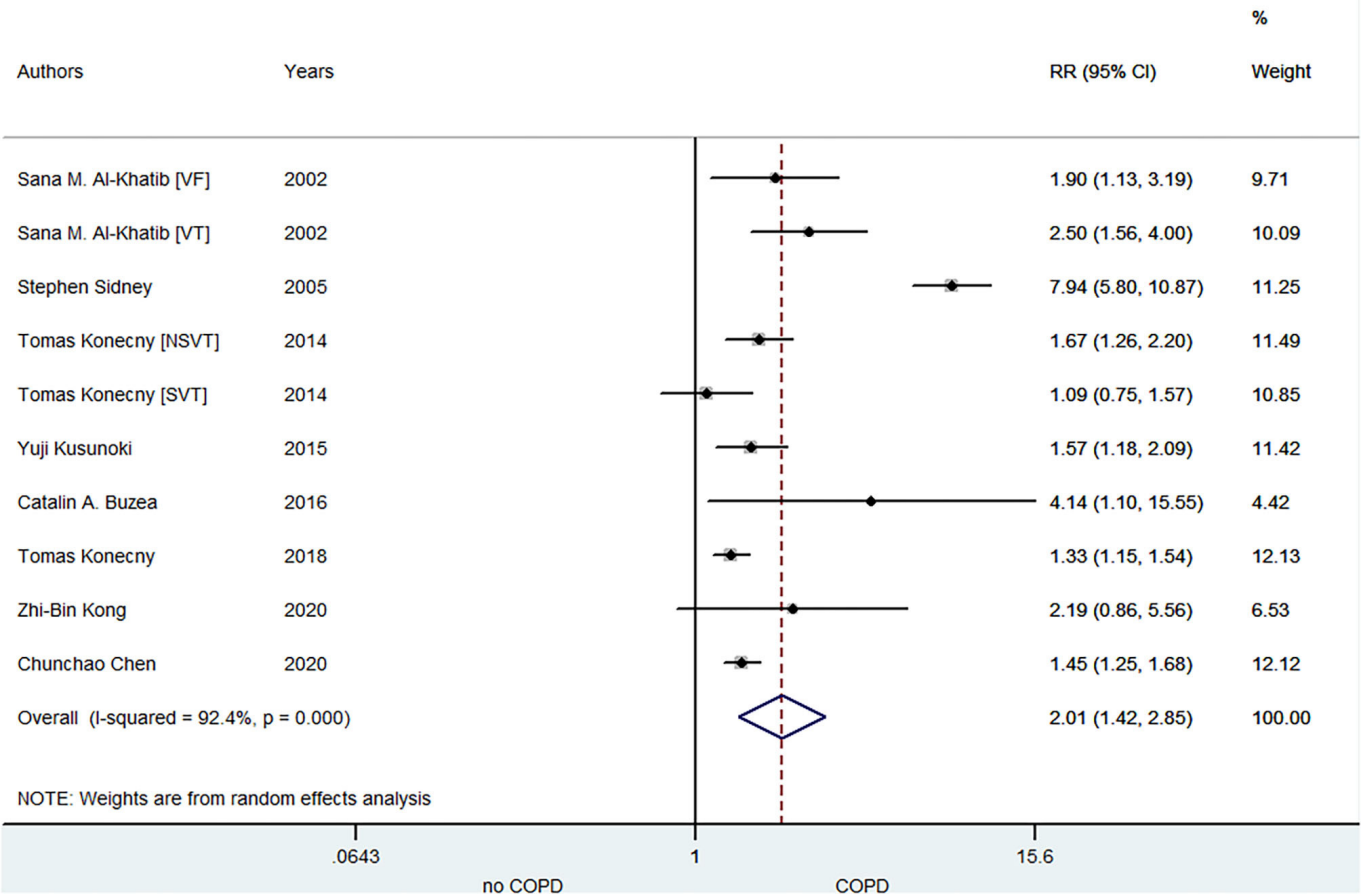
Meta-analysis of the pooled risk ratio of VA in COPD patients. VA, ventricular arrhythmia; RR, risk ratio; VF, ventricular fibrillation; VT, ventricular tachycardia; NSVT, non-sustained ventricular tachycardia; SVT, sustained ventricular tachycardia.

A total of eight included studies focused on the relationship between COPD and VA risk. Konecny et al. (20) demonstrated that there was an independent correlation between the severity of COPD and arrhythmia. Kong (29) indicated this association based on heart rate deceleration runs. Chen (31) manifested the age- and sex-specific association of COPD with VA development. Kusunoki (7) points out that increased VA is likely related to the peculiar pathophysiology of COPD. As shown in Figure 3, the pooled result from the random-effects model showed that COPD was associated with an increased risk of VA (RR = 2.01, 95% CI: 1.42–2.85).

### Relationship Between COPD and SCD Risk

**Figure 4:**
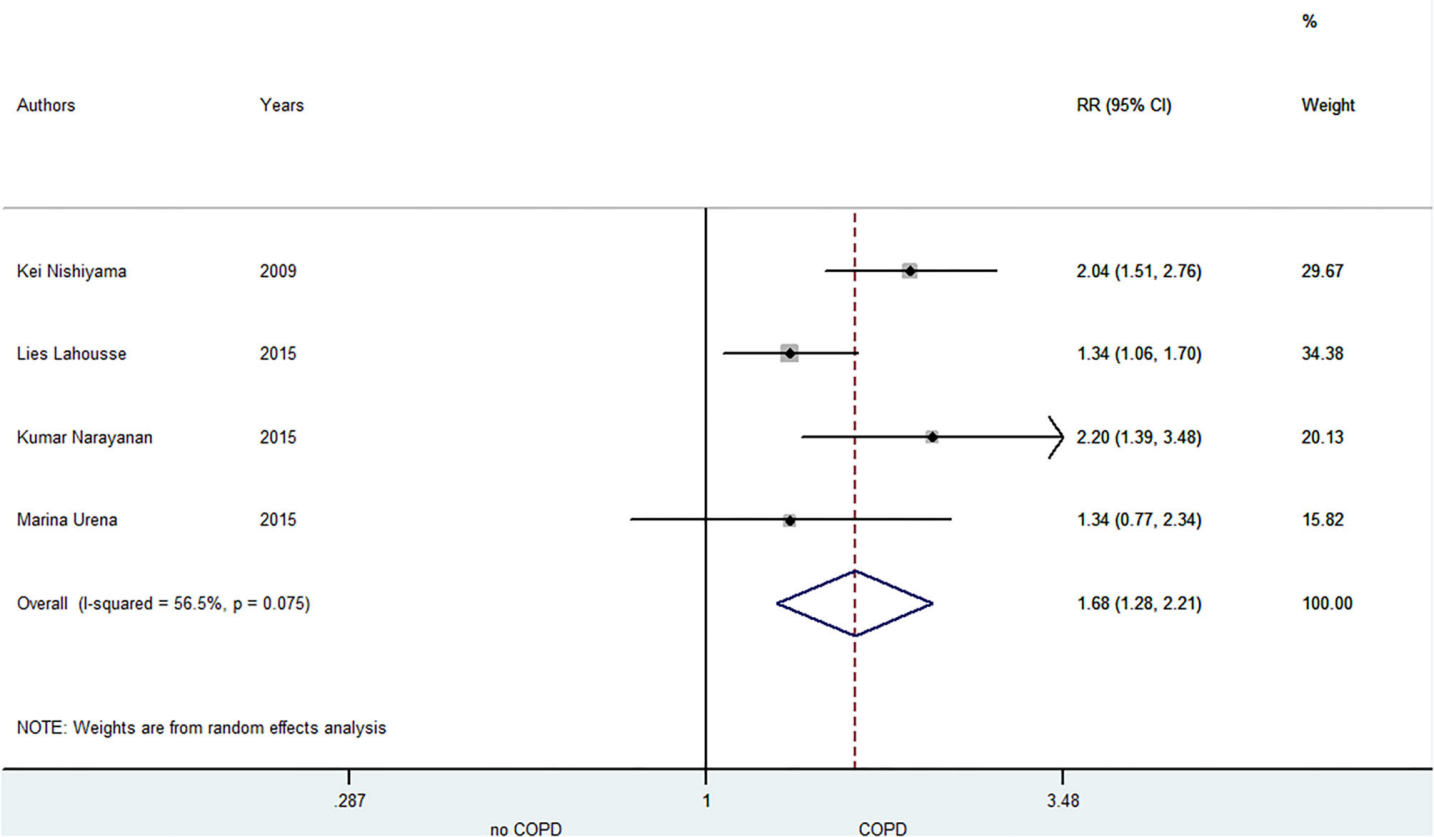
Meta-analysis of the pooled risk ratio of SCD in COPD patients. SCD, sudden cardiac death; RR, risk ratio.

A total of five included studies focused on the relationship between COPD and SCD risk. Lahousse (28) indicated that the risk of SCD especially increased in patients with frequent exacerbations within 5 years of the diagnosis of COPD. Narayanan (11) illustrated that COPD was associated with SCD risk in the community, and the medications, electrocardiographic risk markers and left ventricular ejection fraction did not influence this association. As shown in Figure 4, the pooled result from the random-effects model showed that COPD was associated with an increased risk of SCD (RR = 1.68, 95% CI: 1.28–2.21).

### Sensitivity Analysis

In the sensitivity analysis, the pooled results were stable when we re-performed the above-mentioned analysis using the fixed-effects model (Supplementary Figures 1–3). In addition, the corresponding results were not changed after exclusion one study at a time (Supplementary Figures 7–9).

### Publication Bias

The publication biases assessed by the funnel plots are presented in Supplementary Figures 4–6. No potential publication biases were observed by inspecting the funnel plots. Furthermore, both the Egger's and Begg's tests showed no significant publication biases (all *P* > 0.1).

## Discussion

To the best of our knowledge, this was the first meta-analysis to assess the relationship between COPD and arrhythmia risks. Our results based on the random-effect model analysis demonstrated that COPD was associated with the risks of AF, VA, and SCD. The corresponding results were not changed after exclusion one study at once. The pooled results were also stable when we re-performed the analysis by using the fixed-effects model.

Several studies have shown that COPD is associated with increased risks of cardiovascular diseases (e.g., arrhythmia, ischemic heart disease, heart failure, and arterial disease). Cosgun et al. (35) reported that the markers of arrhythmia (Tp-e, cTp-e intervals, Tp-e/QT ratio, and Tp-e/QTc ratio) were significantly higher in COPD patients compared with non-COPD individuals, suggesting the relationship between COPD and arrhythmia risks. Konecny et al. (27) evaluated the severity of COPD and found that it was an independent risk factor for VA in COPD patients. Cardiovascular diseases and COPD have a pro-inflammatory state characterized by oxidative stress and accelerated aging (36), which are considered to be a possible mechanism for the increased risk of cardiovascular diseases in COPD patients. However, compelling evidence to formally quantified the increased risks of arrhythmia in patients with COPD are still lacked. Herein, we summed up relevant studies to figure out the clear relationship between COPD and arrhythmia risks, suggesting that COPD was associated with the risks of AF, VA, and SCD.

Recent studies have explored the pathophysiological mechanisms between COPD and arrhythmias. As an important pathological manifestation of COPD, hypoxia has an impact on atrial electrophysiology, potentially resulting in the development of arrhythmias. Lammers et al. (37) have reported that hypoxia can cause reentrant arrhythmia in the experimental rabbits particularly at the apex of the heart. Both hypercapnia and hypoxemia caused by COPD can lead to the pulmonary arterioles contracting, which in turn increases pulmonary artery and right atrial pressure. The increased pressure of the right atrium will cause the expansion of the right atrium, the hemodynamic changes of the endocardial blood vessels, and the resetting of blood flow, resulting in the increased arrhythmia susceptibility (38).

Our results in this meta-analysis showed that patients with COPD were more likely to suffer from AF. A large population-based cohort study conducted by Mapel et al. (17) showed the risk of AF in COPD subjects was increased significantly, and patients with COPD might have sustained significant myocardial damage when AF is combined. AF in patients with COPD is more likely to worsen dramatically due to frequent deterioration and enlargement of the left atrium. Moreover, Stevenson illustrated that different recovery of effective refractory period and conduction after hypercapnia may be the reasons for the increased risk of AF in patients with COPD (39). In addition, the ventricular systolic dysfunction (40) and the increased systolic pressure caused by COPD could trigger AF (41), respectively.

We also found that COPD was associated with an increased risk of VA. In patients with COPD, there are a large number of risk factors that induce VA, such as hypoxia, acidosis, and decreased FEV_1_ (42). Mulloy et al. (43) showed that because of alveolar hypoventilation and imbalance of ventilation/perfusion ratio, COPD patients had a significant reduction in oxygen saturation during sleep. Besides, it was found by Shepard et al. (44) that patients with average SaO_2_ <0.80 had a particularly high frequency of VA. Therefore, we indicated that night hypoxia caused by COPD is one of the factors that increase the risk of VA.

In addition, similar results as above were observed in the relationship between COPD and SCD. Several typical and severe features of COPD were also key factors for SCD, including prolonged heart rate, hypoxia, hypoxemia, myocardial ischemia, and heart failure. Lahousse et al. (28) revealed that SCD incidence significantly increased in the case of reduced ventilation and weaken responses to hypercapnia. That's why the night was a time of high incidence of SCD in patients with COPD. Furthermore, the correlation between COPD and SCD was independent in smoking but involved in gender, lung function, QT interval variability, tissue hypoxia, and drug treatment (45). Even though there was no study to assess the common genetic risk factors between COPD and SCD, it may also play a key role in the relationship.

Although a clear relationship between arrhythmia and COPD has been verified in our meta-analysis, the arrangement of COPD patients remains controversial. Konecny et al. (26) demonstrated that preventing the occurrence of COPD may help reduce the incidence of arrhythmia. Major inhalation medications have been shown to precipitate arrhythmia. Since β-adrenergic receptors mediate the distribution of potassium in cardiomyocytes during the action potential, β receptor agonists have a potential for arrhythmogenicity (46). Studies have shown that the new use of short-acting beta agonists and long-acting beta agonists will increase the risk of AF (47). Salpetera et al. noted that a single, regular dose of inhalation of salbutamol enhances atrioventricular nodal conduction and shortens the atrial refractory period, thereby increasing the incidence of arrhythmias (48). However, Chen et al. (31) reported that the combination of short-acting Beta2 agonists and short-acting muscarinic agonists helped reduce VA risk. Since available data could not draw a unanimous conclusion, a more detailed, larger sample study was expected to conduct for investigating a better treatment in COPD patients with arrhythmia.

## Limitations of Study

Overall, our current meta-analysis still had several limitations. First, since most of the selected studies do not perform a bronchial reversibility test to distinguish COPD from asthma, the diagnosis of COPD is not accurate enough. Second, we did not register this research in the PROSPERO (International prospective register of systematic reviews) system. Nevertheless, no similar researches have appeared in this system. Third, although the result yielded statistically significant, clinical heterogeneity was still obvious. Potential causes included the lack of recent studies and the inconsistent definition of COPD. Fourth, we could not perform the subgroup analysis based on the classification of AF (paroxysmal and persistent) due to the limited data. In addition, we did not conduct subgroup analysis due to insufficient original data while some confounding factors (e.g. race, follow-up time, number of participants, and whether be received treatment) may affect the results.

## Conclusion

Available data support that COPD was associated with increased risks of AF, VA, and SCD. Further high-quality studies would be required to confirm the relationship between COPD and arrhythmia risks.

## Data Availability Statement

The original contributions presented in the study are included in the article/Supplementary Material, further inquiries can be directed to the corresponding author/s.

## Author Contributions

XL, SX, ZC, and SL conceived and designed the study. XL, ZC, and SL contributed to acquisition of data, quality assessment, and design of statistical analyses. XL and ZC interpreted the data and wrote the first draft of the study. All authors contributed to critical revision of the report for important intellectual content and approval of the final version to be published.

## Conflict of Interest

The authors declare that the research was conducted in the absence of any commercial or financial relationships that could be construed as a potential conflict of interest.

## Publisher's Note

All claims expressed in this article are solely those of the authors and do not necessarily represent those of their affiliated organizations, or those of the publisher, the editors and the reviewers. Any product that may be evaluated in this article, or claim that may be made by its manufacturer, is not guaranteed or endorsed by the publisher.
