# Supplementary material for: Association of Chronic Obstructive Pulmonary Disease With Arrhythmia Risks: A Systematic Review and Meta-Analysis
Source: Front Cardiovasc Med. 2021 Sep 30;8:732349. doi: 10.3389/fcvm.2021.732349 (PMC8514787; doi:10.3389/fcvm.2021.732349)
Supplement: Supplementary file 1 [file Data_Sheet_1.DOCX]

**Table S1 Summary of the included studies**

| **NO.** | **Study itemes** |
| --- | --- |
| 1 | Li J, Agarwal SK, Alonso A, et al. Airflow obstruction, lung function, and incidence of atrial fibrillation: the Atherosclerosis Risk in Communities (ARIC) study. Circulation. 2014;129(9):971-980. |
| 2 | Mapel DW, Dedrick D, Davis K. Trends and cardiovascular co-morbidities of COPD patients in the Veterans Administration Medical System, 1991-1999. Copd. 2005;2(1):35-41. |
| 3 | Knuiman M, Briffa T, Divitini M, et al. A cohort study examination of established and emerging risk factors for atrial fibrillation: the Busselton Health Study. European journal of epidemiology. 2014;29(3):181-190 |
| 4 | Lip GYH, Skjøth F, Nielsen PB, Larsen TB. Evaluation of the C(2)HEST Risk Score as a Possible Opportunistic Screening Tool for Incident Atrial Fibrillation in a Healthy Population (From a Nationwide Danish Cohort Study). The American journal of cardiology. 2020;125(1):48-54. |
| 5 | Li YG, Bisson A, Bodin A, et al. C(2) HEST Score and Prediction of Incident Atrial Fibrillation in Poststroke Patients: A French Nationwide Study. Journal of the American Heart Association. 2019;8(13): e012546. |
| 6 | Li YG, Pastori D, Farcomeni A, et al. A Simple Clinical Risk Score (C(2)HEST) for Predicting Incident Atrial Fibrillation in Asian Subjects: Derivation in 471,446 Chinese Subjects, With Internal Validation and External Application in 451,199 Korean Subjects. Chest. 2019;155(3):510-518. |
| 7 | Carter P, Lagan J, Fortune C, et al. Association of Cardiovascular Disease With Respiratory Disease. Journal of the American College of Cardiology. 2019;73(17):2166-2177. |
| 8 | Grymonprez M, Vakaet V, Kavousi M, et al. Chronic obstructive pulmonary disease and the development of atrial fibrillation. International journal of cardiology. 2019;276:118-124. |
| 9 | Liang W, Wu Y, Xue R, et al. C(2)HEST score predicts clinical outcomes in heart failure with preserved ejection fraction: a secondary analysis of the TOPCAT trial. *BMC medicine.* 2021;19(1):44. |
| 10 | Buzea CA, Dan GA, Dan AR, et al. Deceleration and Acceleration Capacities in Risk Stratification for Arrhythmias in Patients With Chronic Obstructive Pulmonary Disease. American journal of therapeutics. 2017;24(1):e44-e51. |
| 11 | Narayanan K, Reinier K, Uy-Evanado A, et al. Chronic Obstructive Pulmonary Disease and Risk of Sudden Cardiac Death. JACC Clinical electrophysiology. 2015;1(5):381-387. |
| 12 | Konecny T, Somers KR, Park JY, et al. Chronic obstructive pulmonary disease as a risk factor for ventricular arrhythmias independent of left ventricular function. Heart rhythm. 2018;15(6):832-838. |
| 13 | Konecny T, Park JY, Somers KR, et al. Relation of chronic obstructive pulmonary disease to atrial and ventricular arrhythmias. The American journal of cardiology. 2014;114(2):272-277. |
| 14 | Lahousse L, Niemeijer MN, van den Berg ME, et al. Chronic obstructive pulmonary disease and sudden cardiac death: the Rotterdam study. European heart journal. 2015;36(27):1754-1761. |
| 15 | Kong ZB, Wang XD, Shen SR, et al. Risk Prediction for Arrhythmias by Heart Rate Deceleration Runs in Patients with Chronic Obstructive Pulmonary Disease. International journal of chronic obstructive pulmonary disease. 2020;15:585-593. |
| 16 | Sidney S, Sorel M, Quesenberry CP, Jr., DeLuise C, Lanes S, Eisner MD. COPD and incident cardiovascular disease hospitalizations and mortality: Kaiser Permanente Medical Care Program. Chest. 2005;128(4):2068-2075. |
| 17 | Kusunoki Y, Nakamura T, Hattori K, et al. Atrial and Ventricular Arrhythmia-Associated Factors in Stable Patients with Chronic Obstructive Pulmonary Disease. Respiration; international review of thoracic diseases. 2016;91(1):34-42. |
| 18 | Chen CC, Lin CH, Hao WR, et al. Association between chronic obstructive pulmonary disease and ventricular arrhythmia: a nationwide population-based cohort study. NPJ primary care respiratory medicine. 2021;31(1):8. |
| 19 | Urena M, Webb JG, Eltchaninoff H, et al. Late cardiac death in patients undergoing transcatheter aortic valve replacement: incidence and predictors of advanced heart failure and sudden cardiac death. Journal of the American College of Cardiology. 2015;65(5):437-448. |
| 20 | Nishiyama K, Shizuta S, Doi T, Morimoto T, Kimura T. Sudden cardiac death after PCI and CABG in the bare-metal stent era: Incidence, prevalence, and predictors. International journal of cardiology. 2010;144(2):263-266. |
| 21 | Al-Khatib SM, Granger CB, Huang Y, et al. Sustained ventricular arrhythmias among patients with acute coronary syndromes with no ST-segment elevation: incidence, predictors, and outcomes. Circulation. 2002;106(3):309-312. |

**Table S2 The definition of COPD in included studies**

| **Included studies** | **Definition of COPD** |
| --- | --- |
| Jingjing Li et al. 2014 | GOLD criteria; FEV/FVC <0.70; |
| Douglas W. Mapel et al. 2005 | ICD-9-CM (491-492,496); |
| Matthew Knuiman et al. 2013 | ICD-9 (490–496); |
| Gregory Y.H. Lip et al. 2019 | ICD-10; |
| YanGuang Li-1 et al. 2019 | ICD-10; |
| YanGuang Li-2 et al. 2019 | Medical history; |
| Paul Carter et al. 2019 | ICD-10; OPCS-4; |
| Maxim Grymonprez et al. 2018 | Spirometry; FEV/FVC <0.70; |
| Weihao Liang et al.2021 | Medical record; |
| Catalin A. Buzea et al. 2016 | GOLD criteria; FEV/FVC <0.70; |
| Kumar Narayanan et al. 2015 | Medical record; |
| Tomas Konecny et al. 2018 | Spirometry; FEV/FVC <0.70; |
| Tomas Konecny et al. 2014 | Spirometry; FEV/FVC <0.70; |
| Lies Lahousse et al. 2015 | GOLD criteria; FEV/FVC <0.70; |
| Zhi-Bin Kong et al. 2020 | FEV1 after administration of inhalational bronchodilator; FEV/FVC <0.70; |
| Stephen Sidney et al. 2005 | GOLD criteria; FEV/FVC <0.70; |
| Yuji Kusunoki et al. 2016 | Spirometry; FEV/FVC <0.70; |
| Chun-Chao Chen et al. 2020 | Spirometry; FEV/FVC <0.70; |
| Marina Urena et al. 2015 | Spirometry; FEV/FVC <0.70; |
| Kei Nishiyama et al. 2009 | Medical record; |
| Sana M. Al-Khatib et al. 2002 | Medical record; |

GOLD=Global Initiative for Chronic Obstructive Lung Disease; ICD-9=International Classiﬁcation of Diseases, ninth revision; ICD-9-CM=International Classification of Diseases, 9th Revision, Clinical Modification; OPCS-4=Ofﬁce of Population Censuses and Surveys Classiﬁcation of Interventions and Procedures; FEV1= forced expiratory volume in 1 second; FVC=forced vital capacity;

**Table S3 The definition of AF in included studies**

| **Included studies** | **Definition of AF** | **Permanent AF or paroxysmal AF** |
| --- | --- | --- |
| Jingjing Li et al. 2014 | ICD-9(427.3); ICD-10 (148) | Paroxysmal AF |
| Douglas W. Mapel et al. 2005 | Veteran medical data | Not available |
| Matthew Knuiman et al. 2013 | ICD9-CM (427.31,427.32,427.3) | Not available |
| Gregory Y.H. Lip et al. 2019 | ICD-10 (I48) | Paroxysmal AF |
| YanGuang Li-1 et al. 2019 | ICD-10 | Paroxysmal AF/persistent AF |
| YanGuang Li-2 et al. 2019 | FHS; ARIC; CHARGE-AF; Medical record; | Paroxysmal AF/persistent AF |
| Paul Carter et al. 2019 | ICD-10; OPCS-4; | Not available |
| Maxim Grymonprez et al. 2018 | Laboratory test (ECG) | Paroxysmal AF/persistent AF |
| Tomas Konecny et al. 2014 | medical record or ICD-9 | Not available |
| Stephen Sidney et al. 2005 | ICD-9 (427.31 427.32) | Paroxysmal AF |

Abbreviations: AF=atrial ﬁbrillation; ICD-9=International Classiﬁcation of Diseases, ninth revision; FHS=Framingham Heart Study; ARIC= Atherosclerosis Risk In Communities Study; CHARGE-AF= Cohorts for Heart and Aging Research in Genomic Epidemiology-Atrial Fibrillation; OPCS-4= Ofﬁce of Population Censuses and Surveys, 4th Revision;

**Table S4 The definition of VA in included studies**

| **Included studies** | **Definition of VA** |
| --- | --- |
| Catalin A. Buzea et al. 2016 | Laboratory test (ECG) |
| Tomas Konecny et al. 2018 | Laboratory test or self-report |
| Tomas Konecny et al. 2014 | Medical record or ICD-9 |
| Zhi-Bin Kong et al. 2020 | Laboratory test or self-report |
| Stephen Sidney et al. 2005 | ICD9 (427.1, 427.41, 427.5) |
| Yuji Kusunoki et al. 2016 | Laboratory test or self-report |
| Chun-Chao Chen et al. 2020 | ICD-9 (427.1) |
| Sana M. Al-Khatib et al. 2002 | Sx |

Abbreviations: Sx= signs and symptoms; VA= ventricular arrhythmia;

ICD-9= International Classiﬁcation of Diseases, ninth revision;

**Table S5 The definition of SCD in included studies**

| **Included studies** | **Definition of SCD** |
| --- | --- |
| Kumar Narayanan et al. 2015 | Sx |
| Lies Lahousse et al. 2015 | Sx |
| Marina Urena et al. 2015 | VARC-2 criteria |
| Kei Nishiyama et al. 2009 | Sx |

Abbreviations: SCD= sudden cardiac death; VARC= Valve Academic Research Consortium;

Sx=signs and symptoms;

**Table S6 The adjusting for confounders in included studies**

| **Included studies** | **Adjusting confounders** |
| --- | --- |
| Jingjing Li et al. 2014 | age (continuous); sitting height; sitting height squared; cigarette-years of smoking; |
| Douglas W. Mapel et al. 2005 | age; gender; |
| Matthew Knuiman et al. 2013 | age; height; hypertension treatment; BMI terms |
| Gregory Y.H. Lip et al. 2019 | age; CAD; Hypertension; HF; Thyriod disease; |
| YanGuang Li-1 et al. 2019 | age; HF; CAD; hyperthyroidism; Hypertension; Vascular disease; Diabetes mellitus; gender;  Hyperlipidemia; Renal dysfunction; Valve disease; |
| YanGuang Li-2 et al. 2019 | age; CAD; Hypertension; HF; SHD; Hyperthyroidism; |
| Paul Carter et al. 2019 | age; sex; ethnic group;  the cardiovascular diseases; cardiac procedures;  common causes of death stated earlier; |
| Maxim Grymonprez et al. 2018 | age; sex; pack years of cigarette smoking; additionally stratiﬁed according to exacerbation frequency; left  atrial size; baseline systemic inﬂammatory levels; |
| Weihao Liang et al.2021 | gender; treatment arm; diabetes  mellitus; smoke or ever smoke; BMI; heart  rate; diastolic blood pressure; estimated glomerular  filtration rate; |
| Catalin A. Buzea et al. 2016 | NA |
| Kumar Narayanan et al. 2015 | age; diabetes mellitus; LVEF; medications (including SBA use); smoking; ECG markers; |
| Tomas Konecny et al. 2018 | age; left ventricular ejection fraction; sex; BMI; hypertension; chronic kidney disease; CAD; cancer history; diabetes mellitus; |
| Tomas Konecny et al. 2014 | age; sex; BMI; nicotine dependence; CAD; hypertension; HF; anemia; diabetes mellitus; chronic kidney disease; cancer; |
| Lies Lahousse et al. 2015 | age; sex; pack-years of cigarette smoking; |
| Zhi-Bin Kong et al. 2020 | NA |
| Stephen Sidney et al. 2005 | sex; preexisting CVD study end points; hypertension; hyperlipidemia; diabetes; stroke; |
| Yuji Kusunoki et al. 2016 | NA |
| Chun-Chao Chen et al. 2020 | comorbidities; medication; urbanization level; monthly income; |
| Marina Urena et al. 2015 | NYHA functional; CAD; Prior pacemaker; LVEF; PASP; Stroke; Myocardial infarction; |
| Kei Nishiyama et al. 2009 | age; BMI; Dialysis; Congestive heart failure; Left ventricular dysfunction; CAD; Diabetes with insulin therapy; Chronic renal disease; Peripheral artery disease; Triple vessel disease; |
| Sana M. Al-Khatib et al. 2002 | the significant predictors of mortality |

**Abbreviations:** CAD=Coronary arterial disease; LVEF=left ventricular ejection fraction; CVD=cardiovascular disease; SHD=structural heart disease; SBA=short-acting beta-2 agonist; ECG=electrocardiographic; BMI=body mass index; HF=heart failure; PASP=pulmonary artery systolic pressure;


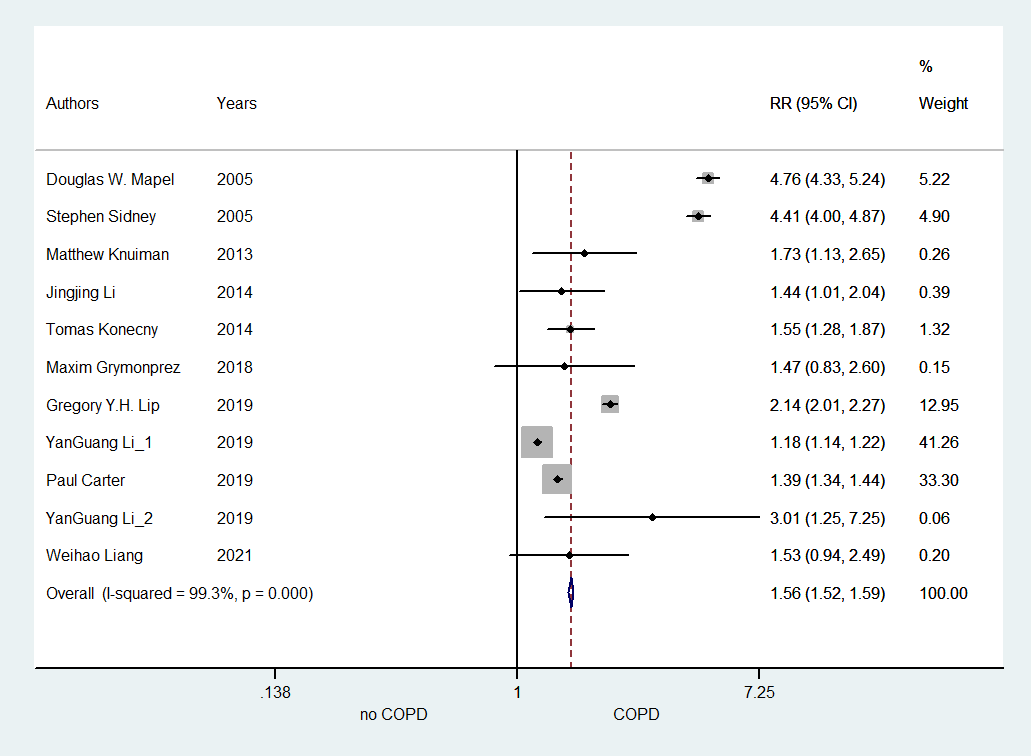
**Figure S1 Analysis using the fixed-effects model for AF in COPD patients**

Abbreviations: AF=atrial ﬁbrillation; RR= risk ratio;

**Figure S2 Analysis using the fixed-effects model for VA in COPD patients**


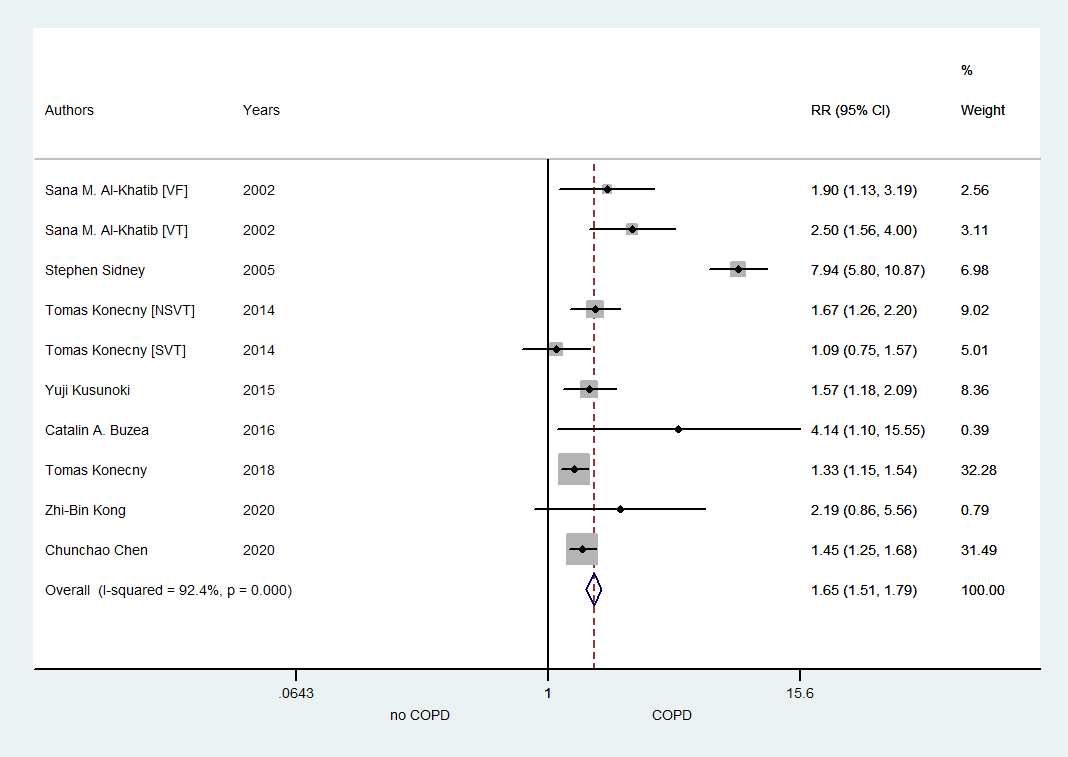


Abbreviations: VA=ventricular arrhythmia; RR=risk ratio; VF=ventricular fibrillation; VT=ventricular tachycardia; NSVT=non-sustained ventricular tachycardia; SVT=sustained ventricular tachycardia;

**Figure S3 Analysis using the fixed-effects model for SCD in COPD patients**


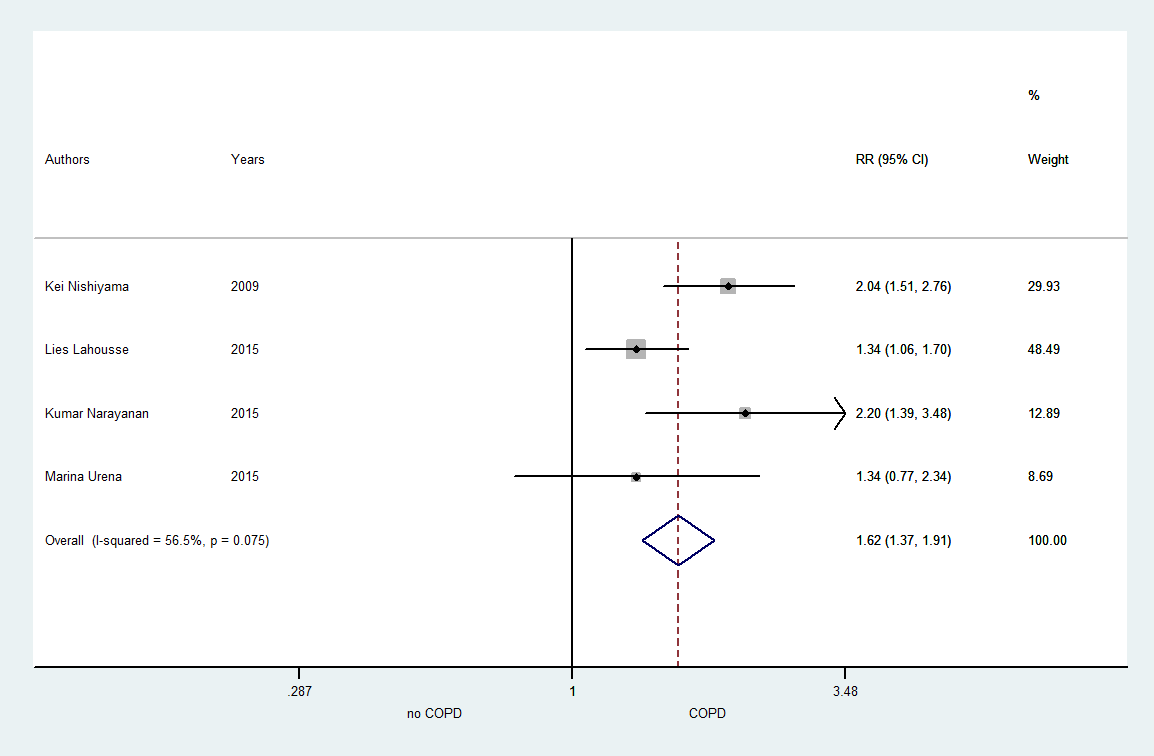


Abbreviations: SCD=sudden cardiac death; RR=risk ratio;

**Figure S4 Funnel plots for AF in COPD patients**


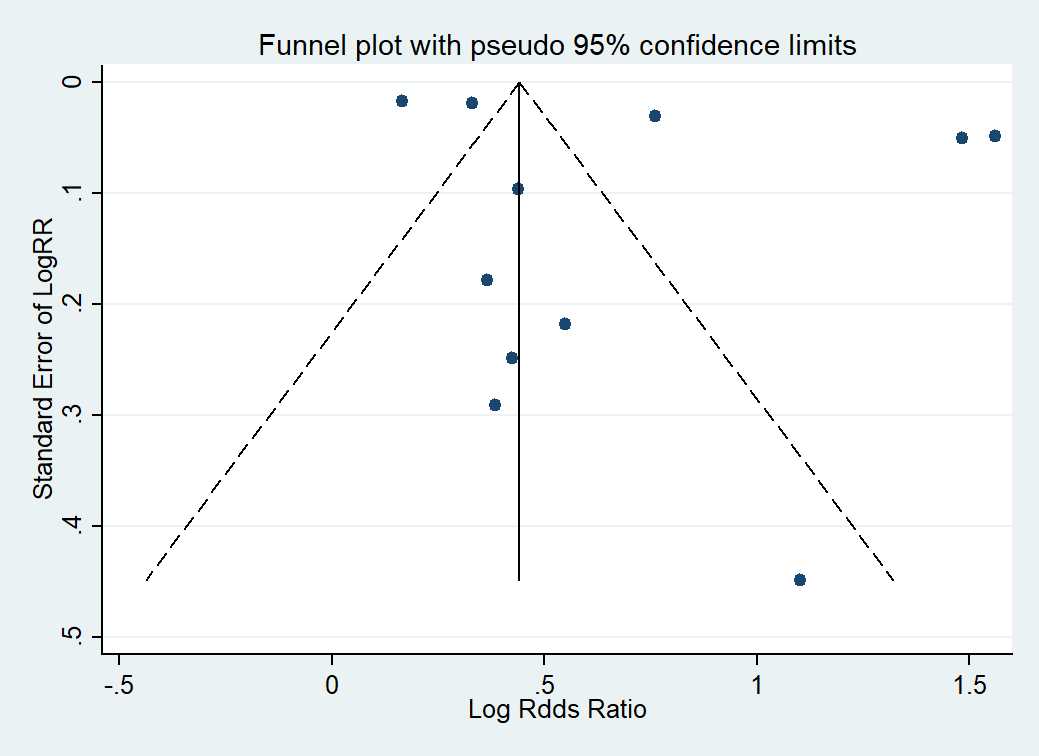


Abbreviations: AF=atrial ﬁbrillation;

**Figure S5 Funnel plots for VA in COPD patients**


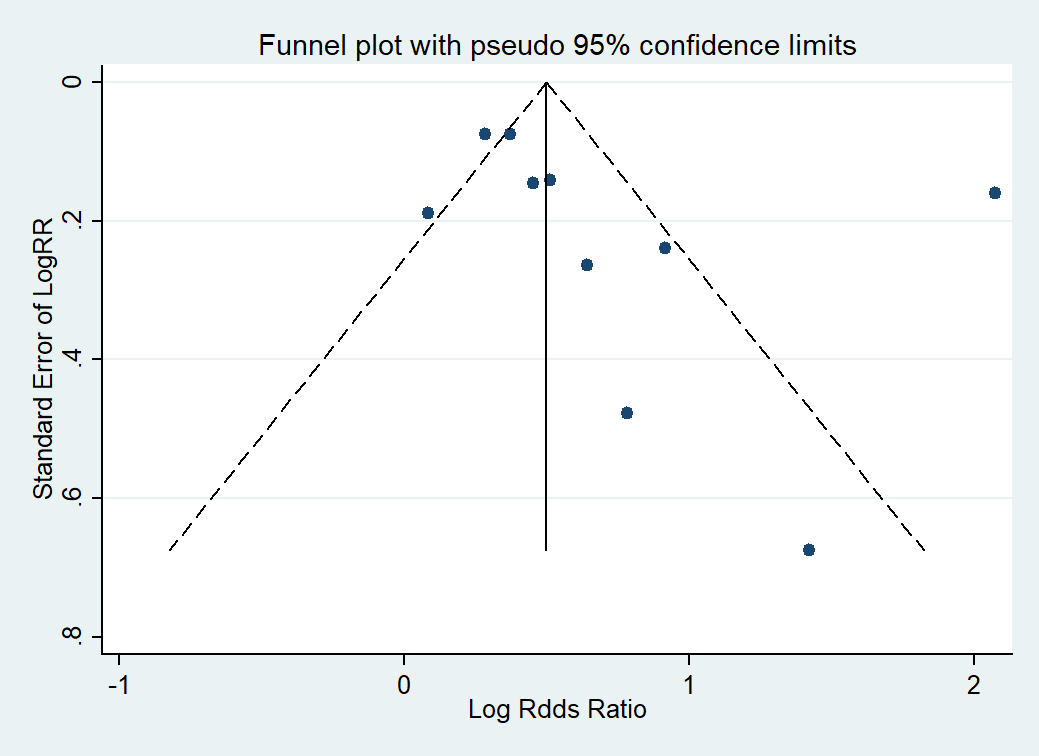


Abbreviations: VA=ventricular arrhythmia; RR=risk ratio; VF=ventricular fibrillation; VT=ventricular tachycardia; NSVT=non-sustained ventricular tachycardia; SVT=sustained ventricular tachycardia;

**Figure S6 Funnel plots for SCD in COPD patients**


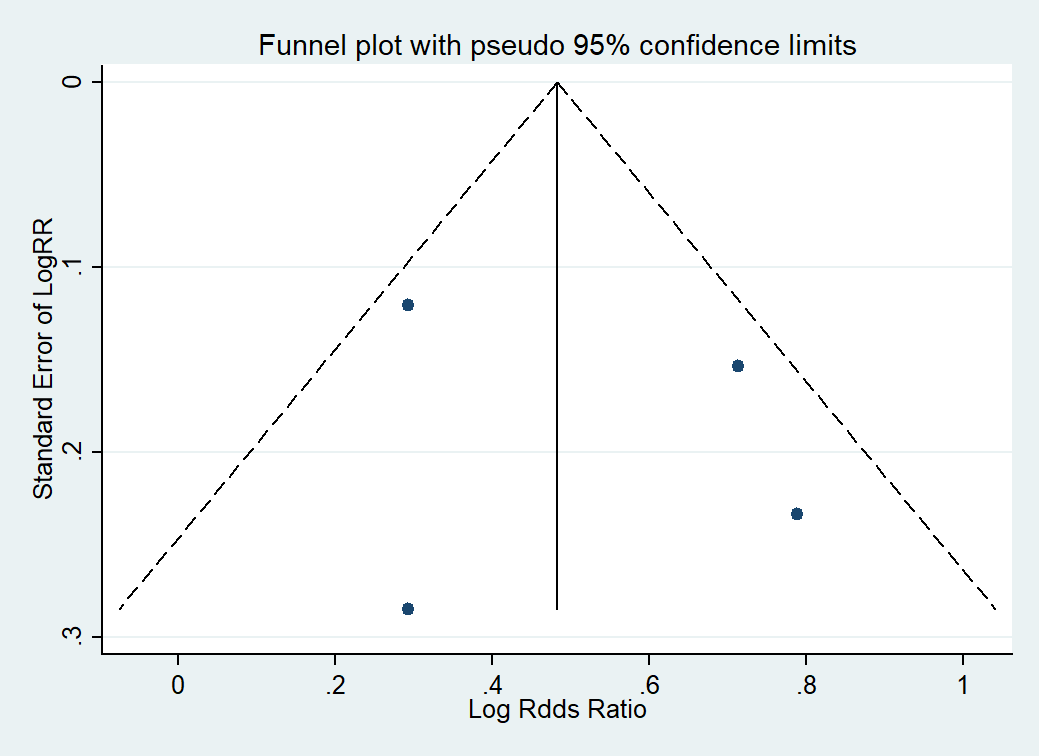


Abbreviations: SCD=sudden cardiac death;

**Figure S7 Sensitivity analysis for AF in COPD patients**


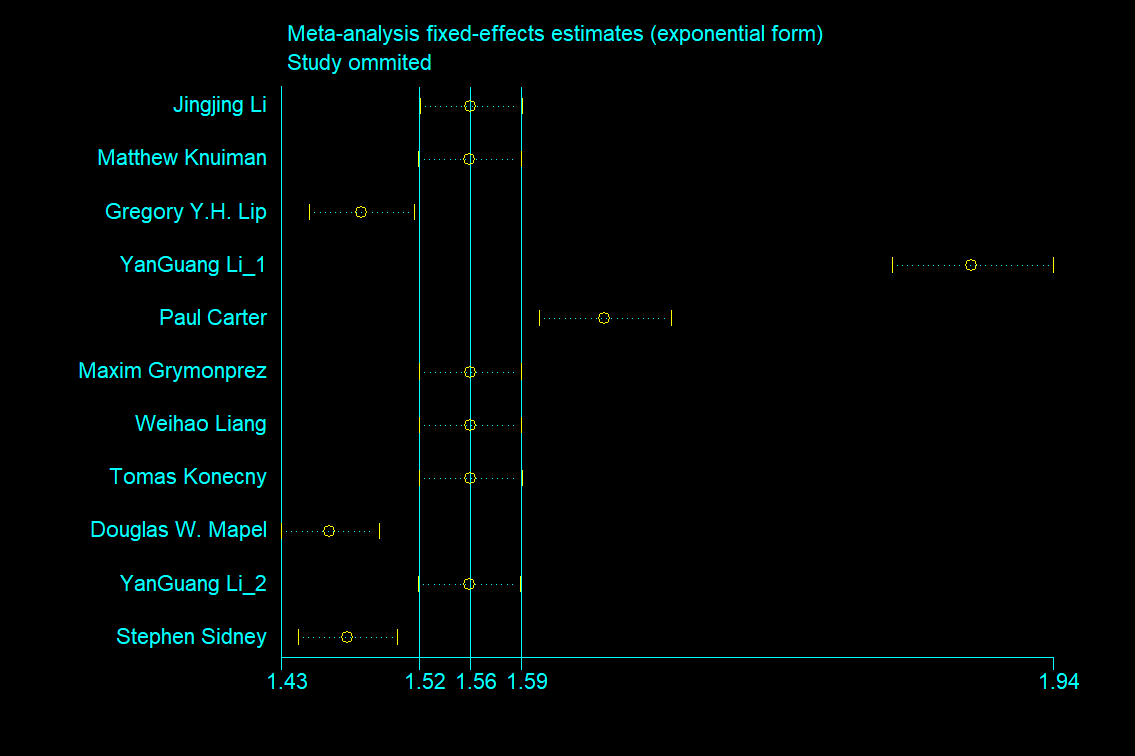


Abbreviations: AF=atrial ﬁbrillation;

**Figure S8 Sensitivity analysis for VA in COPD patients**


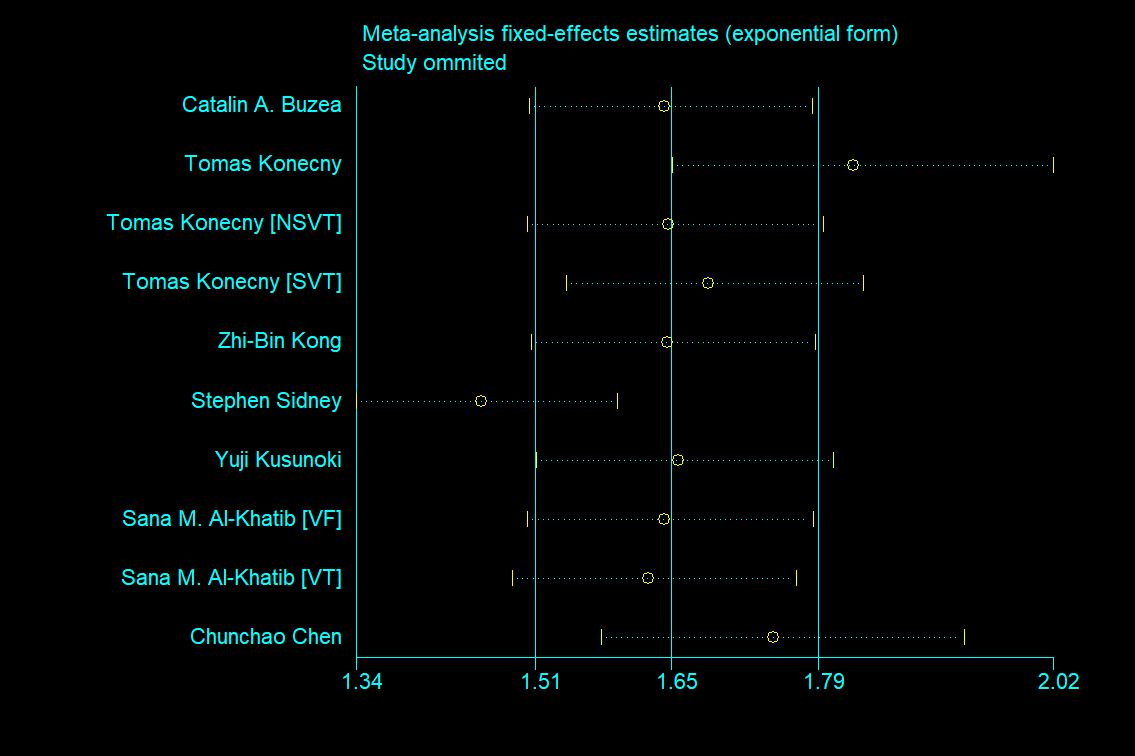


Abbreviations: VA=ventricular arrhythmia;

**Figure S9 Sensitivity analysis for SCD in COPD patients**


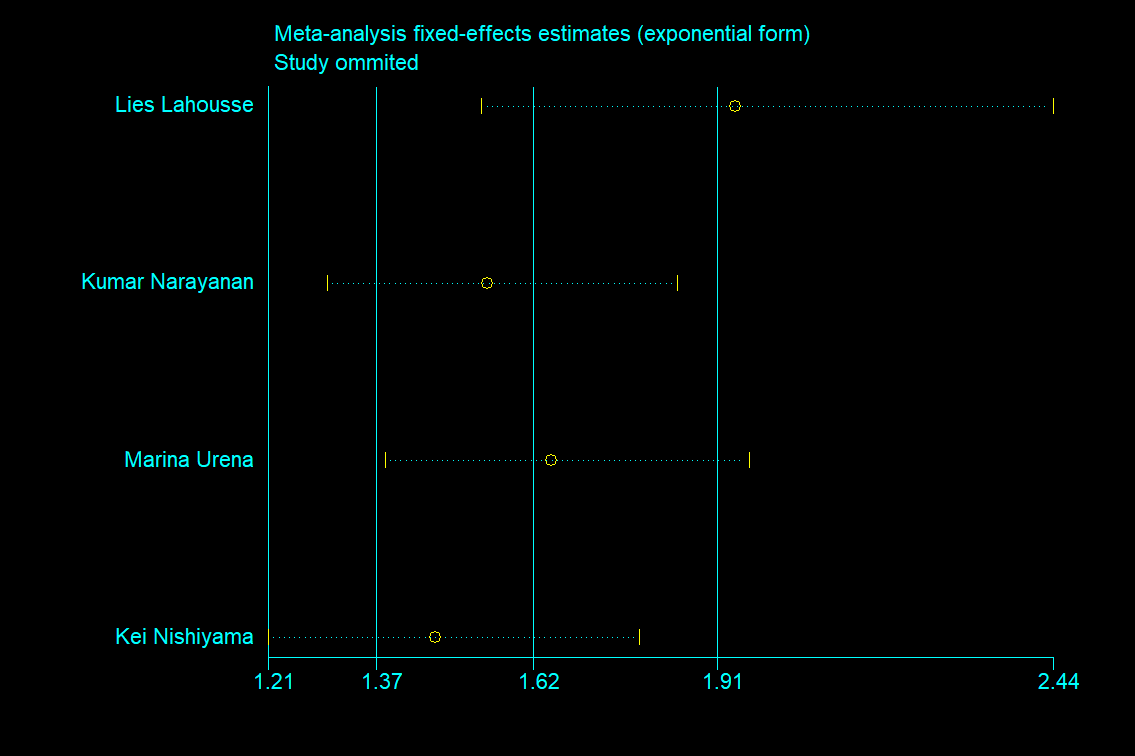


Abbreviations: SCD=sudden cardiac death;
